# Structural and functional characterization of a calcium-activated cation channel from *Tsukamurella paurometabola*

**DOI:** 10.1038/ncomms12753

**Published:** 2016-09-28

**Authors:** Balasundaresan Dhakshnamoorthy, Ahmed Rohaim, Huan Rui, Lydia Blachowicz, Benoît Roux

**Affiliations:** 1 Division of Biological Sciences, Department of Biochemistry and Molecular Biology, The University of Chicago 929 East 57th Street Chicago, Illinois 60637, USA; 2 Department of Biophysics, Faculty of Science, Cairo University, Giza, 12613, Egypt

## Abstract

The selectivity filter is an essential functional element of K^+^ channels that is highly conserved both in terms of its primary sequence and its three-dimensional structure. Here, we investigate the properties of an ion channel from the Gram-positive bacterium *Tsukamurella paurometabola* with a selectivity filter formed by an uncommon proline-rich sequence. Electrophysiological recordings show that it is a non-selective cation channel and that its activity depends on Ca^2+^ concentration. In the crystal structure, the selectivity filter adopts a novel conformation with Ca^2+^ ions bound within the filter near the pore helix where they are coordinated by backbone oxygen atoms, a recurrent motif found in multiple proteins. The binding of Ca^2+^ ion in the selectivity filter controls the widening of the pore as shown in crystal structures and in molecular dynamics simulations. The structural, functional and computational data provide a characterization of this calcium-gated cationic channel.

## Abstract (web summary)

Tetrameric cationic channels specificity is determined by the sequence and structural conformation of their selectivity filter. Here, the authors show that a cationic channel from *Tsukamurella paurometabola* is non-selective due to a Ca^2+^-binding motif within its unusual proline-rich filter.

## Graphical abstract

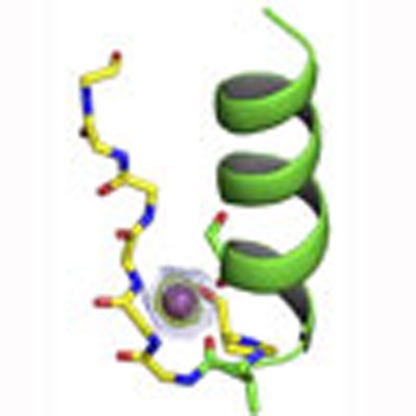

Potassium (K^+^) channels are tetrameric membrane-spanning proteins that provide a selective pore for the conductance of K^+^ across the cell membranes^1^. The X-ray structure of the bacterial channel KcsA from *Streptomyces lividans* revealed that the permeation pathway comprises a narrow pore lined exclusively by main chain carbonyl oxygens^2^. Formed by the residues corresponding to the signature sequence TTVGYG, common to all K^+^ channels^3^,^4^, this essential functional element serves as an ion-conducting selectivity filter, allowing only the passage of nearly dehydrated K^+^ ions. While the X-ray structures of K^+^ channels determined subsequently greatly expanded our knowledge, these efforts have so far revealed only a small number of possible conformations for the selectivity filter. The conformation that is predominantly observed is the ‘ion conductive state'. First revealed by the X-ray structure of KcsA^2^, this conformation of the selectivity filter has been subsequently observed in a number of K^+^ channel pore domains, including KcsA^2^,^5^,^6^,^7^,^8^, Kv1.2 (refs 9, 10), MthK^11^,^12^, KvAP^13^, KirBac^14^, and Kir3.1 (ref. 15). Two additional conformations observed exclusively in crystal structures of the KcsA channel are the ‘constricted' and the ‘flipped' conformations. The constricted or pinched conformation is thought to correspond to a C-type inactivated state of the channel. It has been observed at low [K^+^]^5^, and in the presence of tetraethylammonium, a classic quaternary amine channel blocker^16^, as well as in a series of structures in which the intracellular gate of the channel was open^17^. The flipped conformation has been observed in crystal structures of the non-inactivating mutant E71A^7^,^18^. Thus, only three conformational variants of the selectivity filter have been observed, so far, by X-ray crystallography. In contrast, molecular dynamics (MD) simulations suggest that the selectivity filter of K^+^ channels undergoes considerable thermal fluctuations and can explore a broad range of conformations. Nevertheless, it is difficult to grasp the significance of these computational results because only a limited fraction of the landscape of conformations accessible to the selectivity filter of a K^+^ channel has been mapped out by X-ray crystallography.

Additional information about the structure of related channels with similar sequences can contribute to a better understanding of the conformational landscape of the pore. Implicit in this view is the assumption that the structural diversity displayed by the members of a family of homologous proteins is representative of the structural dynamics displayed by these proteins^19^. In this regard, the conformational variants displayed by the non-specific NaK channel, a distant homologue of K^+^ channels, has helped expand our knowledge of these systems^20^,^21^. Its selectivity filter exhibits extensive structural variability^20^,^21^,^22^,^23^,^24^, converting in response to specific mutations from a fairly wide hydrated pore^20^ to a KcsA-like conductive conformation^22^. An important concept emerging from these studies is that the selectivity filter of tetrameric cationic channels is a complex functional element able to display considerable structural polymorphism and conformational plasticity. While there is an increasing amount of structural data about the broad family of tetrameric 2-transmembrane (TM) pore domains, the extent of the complete conformational landscape of the selectivity filter remains unclear.

Pursuing this strategy, we sought to discover and characterize additional related channels by searching the genomic databases. We specifically focused on 2-TM bacterial channels forming a tetrameric pore domain with a sequence homology below 30% with channels of known structures and with selectivity filters that departed markedly from the highly conserved canonical signature sequence TTVGYGDLMP^3^,^4^. One particular channel from the bacterium *Tsukamurella paurometabola* (Uniprot ID: D5UM26) with an unusual proline-rich selectivity filter sequence, LPMGNGPLSP, attracted our attention. Here, we present a characterization of this cationic channel, hereafter referred to as NaKTs. The crystallographic structure determined at 3.15 Å displays Ca^2+^ ions bound to the channel tetramer within the selectivity filter. Functional assays and electrophysiological recordings show that it is a non-selective cationic channel and that Ca^2+^ binding is necessary for channel activation and gating. The key Ca^2+^ binding motif is formed by an unusual helix-turn structural motif that has not been previously observed in other channels. MD simulations show that Ca^2+^ ions are stable inside the filter and their presence affects the widening of the pore. The gating mechanism involving Ca^2+^ binding in the selectivity filter adds to the molecular repertoire of possible machinery that nature uses to turn channels on and off.

## Results

### Homology to the K^+^ channel family

**Figure 1:**
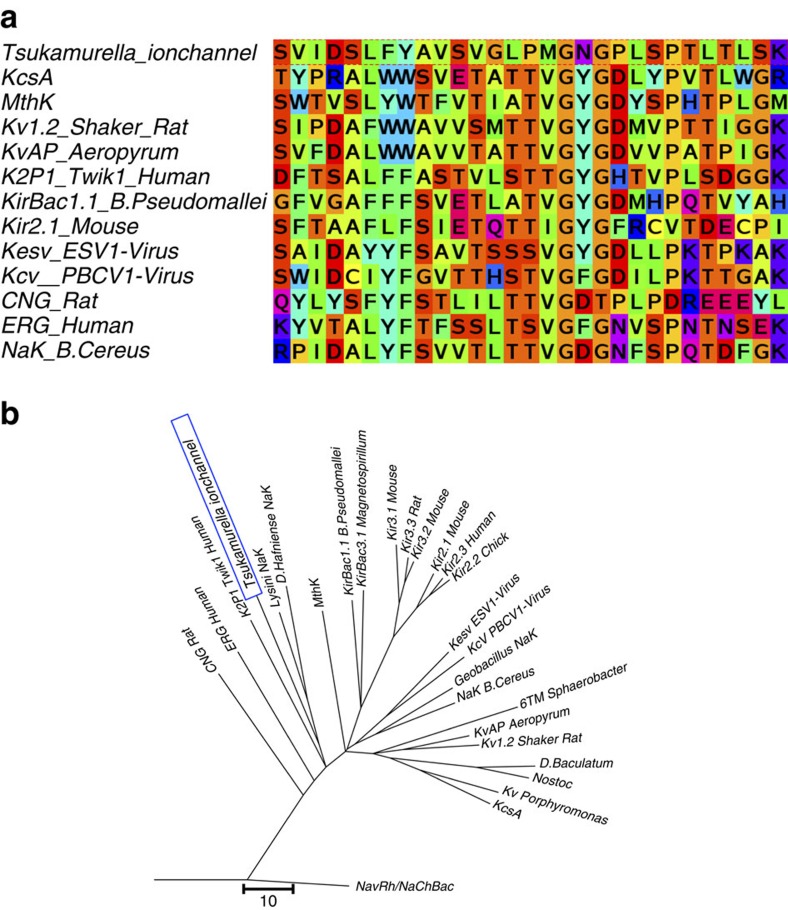
The NaKTs ion channel in relation with other tetrameric cation channels. (**a**) Multiple sequence alignment of the pore helix and selectivity filter regions for the NaKTs ion channel protein along with K^+^, Na^+^ and non-selective channels. The multiple sequence alignment was done using Clustalw and JalView (the colour coding is based on the Taylor scheme available in JalView). (**b**) Phylogenetic tree (scored from pore helix and filter region). The phylogenetic tree was generated based on the multiple sequence alignment of the pore helix and the selectivity filter region only using the programs JalView and MEGA 5.

The NaKTs channel belongs to the family of channels with fourfold symmetric tetrameric pore domains. The subunits display a simple 2-TM topology, M1-pore-M2, in which a long pore loop is located between two TM helices. The selectivity filter of the NaKTs channel is formed by the uncommon proline-rich sequence, LPMGNGPLSP. A search of the non-redundant database (NCBI and UNIPROT) did not discover other channels with a similar sequence, suggesting that this feature is fairly specific to this particular channel. Despite the difference, the NaKTs ion channel is nonetheless clearly a member of the broad K^+^ channel family, with a high homology to many important channels, as shown by the multiple sequence alignment of the NaKTs channel and other K^+^ channel family members (Fig. 1a). The pore region of NaKTs has 30% sequence similarity with that of NaK, a channel that can be mutated into a KcsA-like conductive pore. A phylogenetic tree generated by considering only the sequence of the pore loop region shows the close relationship of NaKTs to the members of the K^+^ channel family (Fig. 1b). On the basis of this analysis, it appears that the pore region of the NaKTs channel is most similar in terms of primary sequence to that of the CNG, hERG, K2P, MthK and Kir channels. This overall similarity suggests that the conformational variants adopted by NaKTs are likely to be of relevance for the entire K^+^ channel family.

### Structure determined by X-ray crystallography

**Figure 2:**
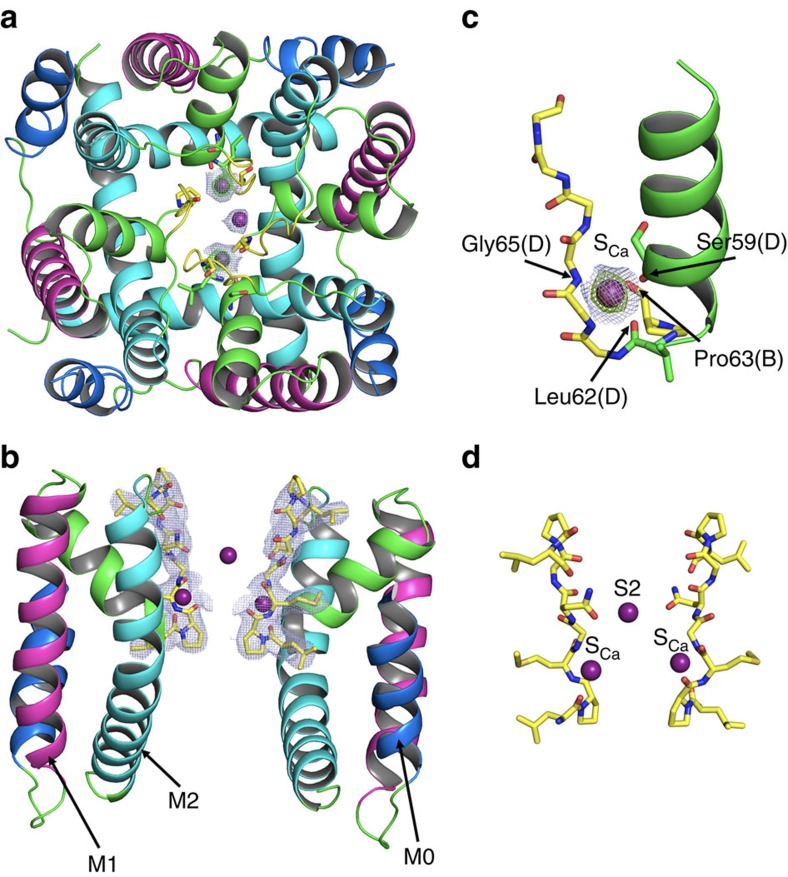
X-ray crystal structure of the NaKTs ion channel determined at 3.15 Å resolution. (**a**) Ribbon representation of top view of the NaKTs tetramer (chain ABDE—dataset 2), along with bound Ca^2+^ ions. The M0 helix (residues 5–19) is shown in blue, the M1 outer helix (residues 25–45) is shown in magenta, the pore helix (residues 46–61) is shown in green, the selectivity filter (residues 62–69) is shown in yellow, the M2 inner helix is shown in cyan and the loop regions are shown in green. Calcium ions are shown as purple spheres, with the 2*F*_o_−*F*_c_ map (grey) at 1σ contour and *F*_o_−*F*_c_ map (green) at 4.5σ contour. (**b**) The TM region of two opposing monomers of the NaKTs ion channel protein (chain A, chain D) shown with the 2*F*_o_−*F*_c_ density map at 1σ contour for the selectivity filter region (yellow sticks). (**c**) *F*_o_−*F*_c_ map (green) at 4.5σ contour and 2*F*_o_−*F*_c_ map (grey) at 1σ contour showing the location of Ca^2+^ ion (purple sphere) at site S_Ca_. The pore helix (green cartoon), the selectivity filter residues (yellow sticks) and Ca^2+^ coordinating AA's are labelled accordingly. (**d**) Close-up view of two opposing monomers of the NaKTs selectivity filter (chain A, chain D) shown in yellow sticks with bound Ca^2+^ ions at sites S2 and S_Ca_.

The crystallographic structure of the NaKTs channel determined at 3.15 Å resolution is shown in Fig. 2. The structural model is complete over all regions, except for residues 1–3 at the N-terminal and residues 107–123 at the C-terminal regions (the latter might have been cleaved by chymotrypsin digestion). In its broad features, the structure of the NaKTs channel is similar to that of other tetrameric pore domains, with a TM helical segment-labelled M1 (residues 21–45), a pore helix (residues 49–62), the selectivity filter (residues Pro63-Met64-Gly65-Asn66-Gly67-Pro68) and a M2 helix (residues 73–101). In addition, an amphipathic helical segment-labelled M0 (residues 5–19) is located at the N-terminal end of the subunit. The channel is in a conformation with an open intracellular gate, with a strong bend of ∼50° near the centre of the M2 helix (Fig. 2b). While the open conformation of NaKTs is similar to that observed in previous X-ray structures of other channels^11^, the strongest bend in the M2 helix occurs at the level of residue Val87 rather than at the level of a glycine residue proposed to act as a conserved gating hinge in several channels (Supplementary Fig. 1). The narrowest point along the intracellular vestibule occurs at the level of residue Val92, its side chain pointing towards the central channel axis. The diameter is on the order of 10 Å, which is slightly smaller than that observed in other channels in an open conformation, for example, NaK (10.3 Å at Phe92) and MthK (12.9 Å at Ala88)^21^. The M2 helix also displays a twist of ∼12° with respect to the open conformation of the NaK channel (3E86), but of only ∼3° with respect to the open conformation of the MthK channel (4HYO) (Supplementary Fig. 2). These structural differences regarding the intracellular activation gate suggest that some aspect of the gating mechanism of NaKTs may differ from other channels. Furthermore, an amphipathic helical M0 segment (residues 5–17) docks against the hydrophobic surface of the pore domain. In this configuration, the N terminus of the protein reaches across the membrane region to interact with Phe41 from a neighbouring subunit near the extracellular side. It seems unlikely that M0 affected the overall geometry of the pore, as it is very similar to that of other tetrameric channels. While this orientation is stabilized by a number of interactions (Supplementary Fig. 3), it is probably not representative of a membrane-bound conformation of the channel. In MD simulations including the M0 segment, water molecules rushed into the membrane interior along the pore domain to solvate charged residues (Lys9 and Arg10). Most likely, the amphipathic M0 segment should be parallel to the membrane interface^25^.

### Cation conducting pore

**Figure 3:**
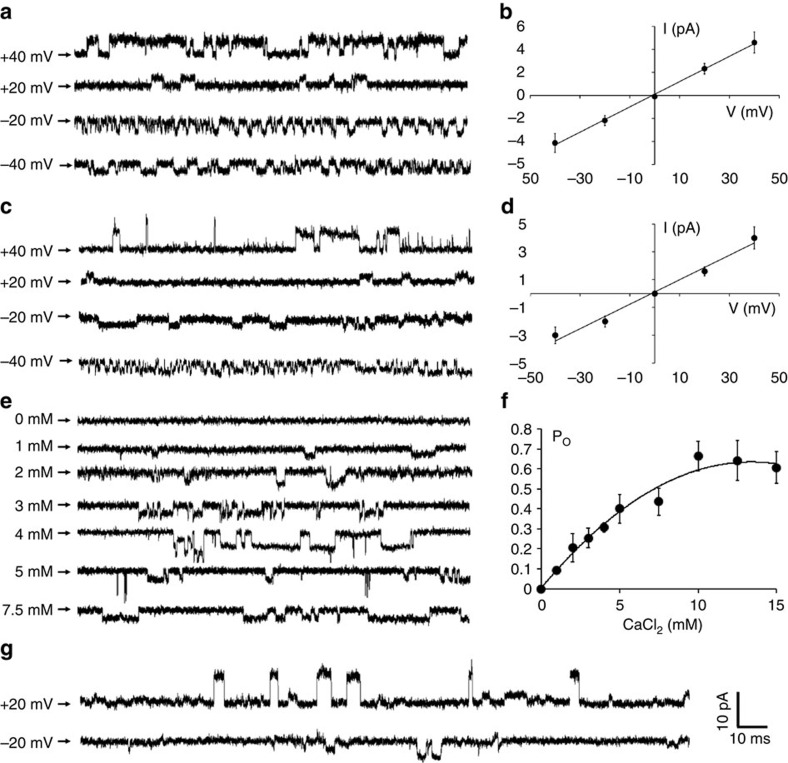
Results from single-channel recordings. (**a**) Representative current traces of NaKTs ion channel Na^+^ conductance at different TM potentials (internal solution: 20 mM HEPES, pH 7.5; 0.2 M NaCl and external solution: 20 mM MOPS, pH 4; 0.2 M NaCl; 5 mM CaCl_2_). (**b**) *I*/*V* relationship of NaKTs ion channel in the presence of NaCl. The NaKTs ion channel Na^+^ conductance is 110 pS. (**c**) Representative current traces of NaKTs ion channel K^+^ conductance at different TM potentials (internal solution: 20 mM HEPES, pH 7.5; 0.2 M KCl and external solution: 20 mM MOPS, pH 4; 0.2 M KCl; 5 mM CaCl_2_). (**d**) *I*/*V* relationship of NaKTs ion channel in the presence of KCl. The NaKTs ion channel K^+^ conductance is 88 pS. (**e**) Representative current traces of the NaKTs ion channel Na^+^ conductance as a function of Ca^2+^ in the external solution at a fixed membrane potential of −40 mV. (**f**) Open-channel probability of NaKTs ion channel, Po, as a function of Ca^2+^ in the external solution at a holding membrane potential of +40 mV (internal solution: 20 mM HEPES, pH 7.5; 0.2 M NaCl and external solution: 20 mM MOPS, pH 4; 0.2 M NaCl with CaCl_2_ ranging from 250 μM to 20 mM). The error bars indicate the mean-square deviation from the average (fraction of open time over a total time interval) based on 3–5 separate time intervals. (**g**) Representative current traces of the chimeric NaKTs-SF_KcsA_ channel at 200 mM KCl with 0 mM CaCl_2_. The single-channel conductance of the chimera is on the order of 300 pS.

The ion-conducting pore of NaKTs is aligned with the fourfold symmetry axis of the tetramer, consistent with the structure of other channels in the family^2^. However, the selectivity filter is formed by four extended polypeptide chains, each containing a proline-rich sequence LPMGNGPLSP that is not commonly observed in other homologue channels (Figs 1 and 2d). The overall topology of the pore loop is similar to that of other tetrameric channels (Supplementary Fig. 4) although there are noticeable structural differences with other members of the tetrameric channel family (Supplementary Table 1). A superimposition of the pore region (that is, the pore helix and the filter) of the NaKTs channel with that of the KcsA channel in the conductive state (PDB id 1K4C) yields a root-mean-square deviation (RMSD) of 2.43 Å for the backbone atoms. The RMSD with other K^+^-selective channels (MthK, Kir2.2, Kir3.2, Kv1.2) is also on the order of 3 Å. For comparison, the RMSD among these various channels is typically much smaller, on the order of 0.5–1.0 Å. This shows that the conformation of the pore region for the NaKTs channel departs from that of other members of the tetrameric channel family. Despite these differences, NaKTs is a non-selective cationic channel according to electrophysiological recordings of the channel reconstituted in lipid membranes. The chymotrypsin-treated form of the channel that was crystallized conducts both K^+^ and Na^+^, with a measured conductance of approximately 88 and 110 pS at 200 mM of KCl and NaCl, respectively (Fig. 3a–d).

### Presence of Ca^2+^ in the selectivity filter

**Figure 4:**
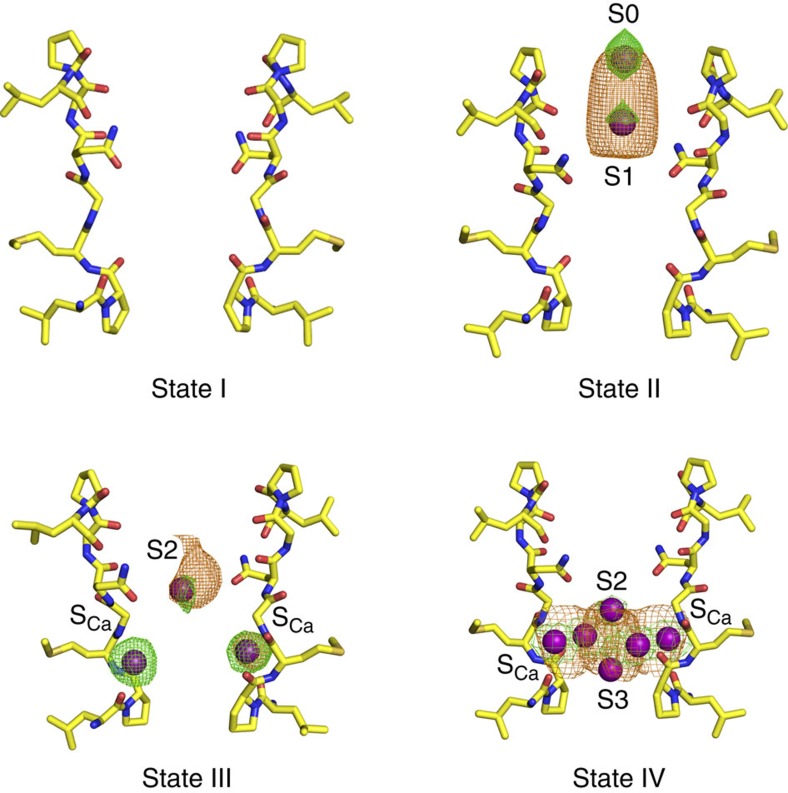
Occupancy states of the selectivity filter of NaKTs. Snapshots of the selectivity filter of the NaKTs ion channel structure (yellow sticks), describing the location of Ca^2+^ ions along the ion-conduction pathway, categorized into four different states. Selectivity filter of state I is seen in three tetramers (chain CGHI of datasets 1 and 3 and chain FJKL of dataset 1), selectivity filter of state II is seen in one tetramer (chain FJKL of dataset 3), selectivity filter of state III is seen in 2 tetramers (chain ABDE of datasets 1 and 2), selectivity filter of state IV is seen in two tetramers (chain ABDE of dataset 3 and chain FJKL of dataset 2). Calcium ions are shown as purple spheres, along with their respective anomalous density maps (orange) at 5–10σ contour and *F*_o_−*F*_c_ map (green) at 4σ contour.

A salient difference between the NaKTs channel and other members of the tetrameric channel family is the presence of Ca^2+^ ions, which are observed in different regions of the selectivity filter. Three different diffraction datasets were analyzed to identify different ion occupancy states (by virtue of the I4 space group symmetry, the six monomers yield three crystallographically related tetramers). A total of nine crystallographically distinct tetramers were considered in the analysis, revealing four different occupancy states, I to IV, with 0, 2, 3 and 6 Ca^2+^ ions, respectively (Fig. 4).

There are no ions in state I, which serves as the reference for the unoccupied pore. In state II, there are two Ca^2+^ ions near the extracellular side in locations corresponding to sites S0 and S1 in KcsA. The Ca^2+^ ion in site S0 is surrounded by four water molecules and does not interact directly with backbone oxygen atoms. The distance between the carbonyl oxygen of Gly67 and the Ca^2+^ ion at site 1 is about 4.9 Å, suggesting that the ion binds in this site in a hydrated form through bound water molecules.

In state III, two Ca^2+^ ions are bound diagonally opposite to each other 4.1 Å away from the pore axis at a site in the selectivity filter referred to as S_Ca_ (Fig. 2c), where they are coordinated by the backbone carbonyl oxygen atoms of Ser59 (distance of 2.6 Å), Leu62 (distance of 2.5 Å) and the backbone nitrogen atom of Gly65 (distance of 2.9 Å) from one subunit, and the backbone oxygen atom of Pro63 from a neighboring subunit (distance of 2.9 Å). A third Ca^2+^ is located at the centre of the pore in a site corresponding to S2 in KcsA. The ion is coordinated by the side chain of Asn66, which points towards the pore of the selectivity filter. The OD1 atom of Asn66 interacts with the Ca^2+^ ion and the ND2 atom of Asn66 interacts with the backbone carbonyl oxygen atom of Leu69. In state IV, there are four Ca^2+^ ions in S_Ca_ and two additional Ca^2+^ ions, one in the site corresponding to S3 in KcsA near Gly65 and a second ion near Pro63 (a different sub-state is seen with the ion position shifted by 2.1 and 1.1 Å towards the intracellular surface). Lastly, there is a tetramer with a single Ca^2+^ ion located at the centre of the intracellular vestibule with no ions observed in the pore, as observed in the KcsA and NaK structures, assigned to State I. More details about the locations of Ca^2+^ ions in the 3.4 Å dataset 3 (3.4 Å resolution) and 3.6 Å dataset 1 (3.6 Å resolution) crystal structures are provided in Supplementary Fig. 5. The Ca^2+^ ion occupancy along the selectivity filter pore and S_Ca_ site were refined using SAD anomalous data and their respective ion occupancy is given in Supplementary Table 2.

### Structural and functional response to the binding of Ca^2+^

**Figure 5:**
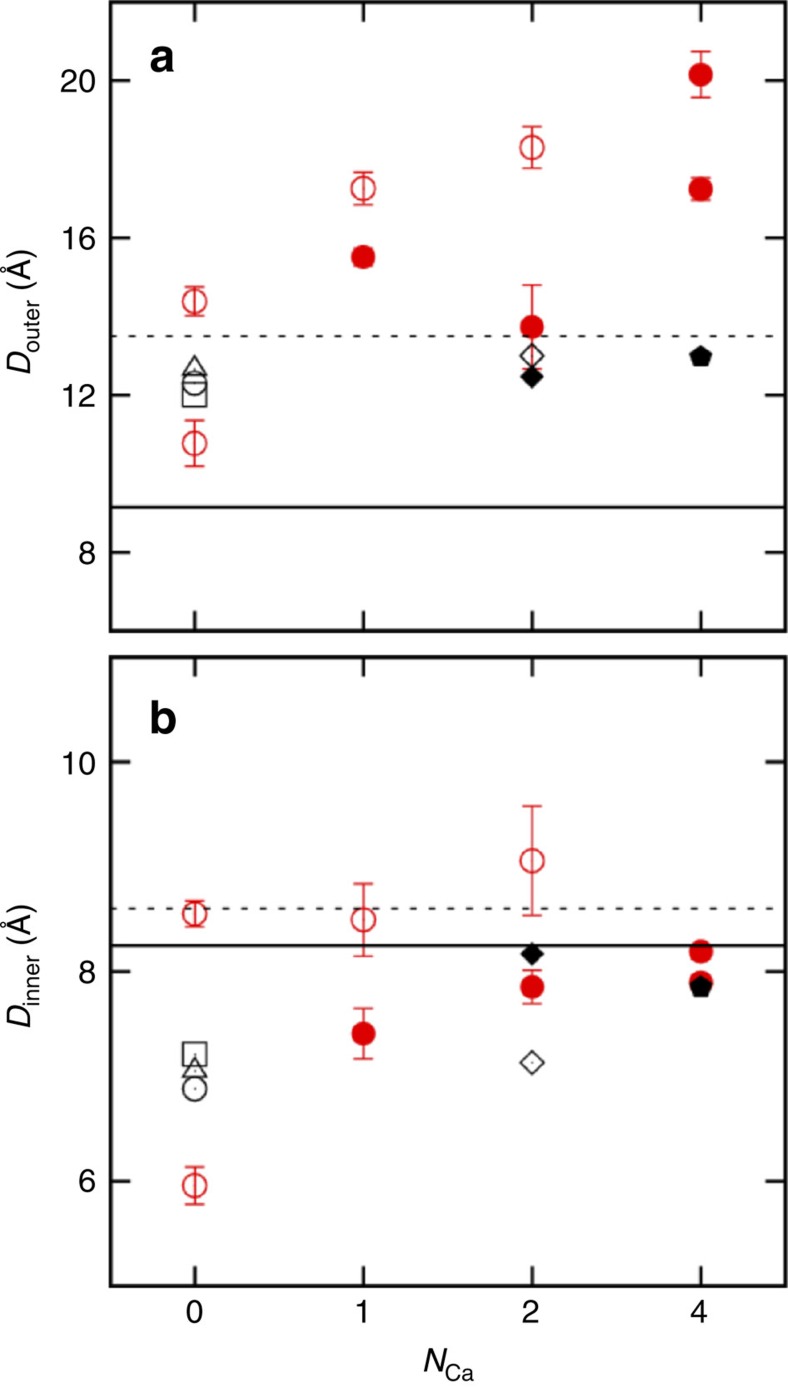
Outer and inner diameters of the selectivity filter pore in the NaKTs channel. (**a**) The outer diameter *D*_outer_ taken as the Cα–Cα distance between the diagonally placed Pro68 is shown. (**b**) The inner diameter *D*_inner_ taken as the Cα–Cα distance between the diagonally placed Pro63 is shown. Supplementary Fig. 4 shows the relative positions of the proline residues. The diameters from the NaKTs channel crystal structures are presented by black symbols. They are grouped by the number of Ca^2+^ at the selectivity filter S_Ca_ site. States I (○ and ▵) and II (□) have no Ca^2+^ at the S_Ca_ site. Two structures are used for State I. The difference between the two lies in that the second structure (▵) has a Ca^2+^ in the centre of the intracellular vestibule. State III (⋄ and ♦) has two Ca^2+^ at the diagonally place S_Ca_ sites; State IV (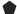 has all four S_Ca_ sites occupied. The averaged *D*_outer_ and *D*_inner_ in the MD simulations (−500 mV) are plotted with red symbols. The error bars indicate the standard deviations of the distances calculated from the trajectories. The distances are calculated from two pairs of diagonally placed subunits. In all cases, filled symbols indicate that the distance is taken from subunits with the S_Ca_ sites occupied by Ca^2+^. The *D*_outer_ and *D*_inner_ in the NaK channel (2AHY, solid line) and KcsA (1K4C, dotted line) are shown as references.

The crystallographic and MD data indicate that the diameter of the pore at its inner and outer ends, defined respectively from the backbone Cα–Cα distance of residues Pro63 and Pro68 from diagonally-opposing subunits, is sensitive to occupancy by Ca^2+^ ions in the pore (Fig. 5). The diameter of the inner pore increases by 1–1.2 Å in response to the binding of a Ca^2+^ ion at site S_Ca_, in the vicinity of Pro63, Gly65, Ser59 and Leu62. Once a Ca^2+^ ion binds to this position, Pro63 sways by about 0.5 Å away from the ion-conduction pathway. If one assumes that widening the selectivity filter transforms the channel from a non-conductive (or poorly conductive) state to a conductive state, Pro63 thus appears as a key residue responsible for channel activation upon the binding of Ca^2+^ at the S_Ca_ sites. Of relevance to these observations, functional measurements of the channel reconstituted in lipid bilayers show that Ca^2+^ is absolutely required for ion-conduction activity, with a *K*_1/2_ of ∼4 mM determined from the open-channel probability (Fig. 3e,f). The observed mean open-channel probability appears to be slightly sensitive to the membrane potential (around 0.25 at negative voltages and 0.6 at positive voltages with 2.5 mM [Ca^2+^]). The average single-channel conductance is not significantly affected by the presence of Ca^2+^.

To probe the functional significance of the S_Ca_ binding site on channel activation, a chimera of the NaKTs channel with its selectivity filter (PMGNGP) replaced by the corresponding residues from the KcsA channel (TVGYGD) was engineered and purified for functional assays. Whereas no channel activity can be observed with the wild-type NaKTs unless Ca^2+^ is present (Fig. 3e), it is observed that this chimeric NaKTs-SF_KcsA_ channel conducts K^+^ in the absence of any Ca^2+^ (Fig. 3g). This observation strengthens the conclusion that the binding of Ca^2+^ at the site S_Ca_ is directly implicated in the activation of the NaKTs channel.

Comparison with other channels shows some similarities but also important differences. A Ca^2+^-dependent gating located within the selectivity filter was also shown to control the K^+^ conduction in the MthK channel^26^. However, while the sequence similarity of NaKTs and MthK is high (Fig. 1a), the latter does not possess the proline (Pro63) that forms part of the S_Ca_ binding site observed in NaKTs and the filter gating observed in the MthK channel is believed to be allosterically triggered by the binding of Ca^2+^ to the cytoplasmic RCK domains at the intracellular side^26^. A recent study based on chimeric channels that replaced the selectivity filter of the bacterial non-selective cation channel NaK, TVGDGNFS, with a cyclic-nucleotide gated channel filter sequence, TIGETPPP also displayed Ca^2+^ block (∼14 μM)^27^. However, there are important differences because the key prolines in those chimeras were located toward the extracellular end of the filter, whereas the S_Ca_ binding site in NaKTs is located at its intracellular end.

### Structural similarity of the S_Ca_ site with other systems

**Figure 6:**
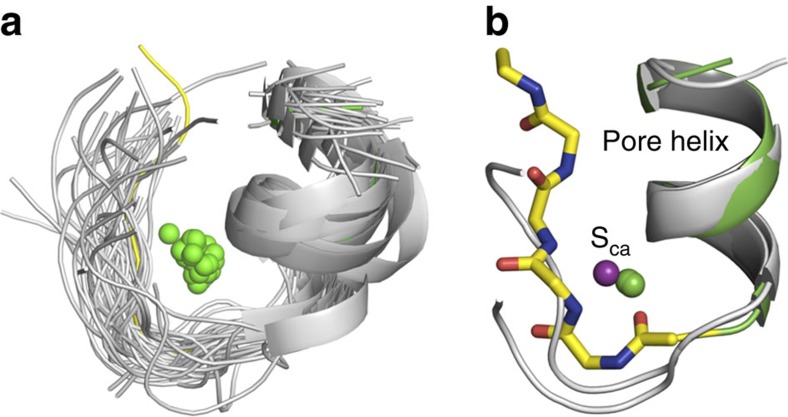
The helix-turn S_Ca_ binding motif (**a**) Superimposition of the Ca^2+^ binding motif of 47 PDB structures (grey cartoon) out of 369 hits obtained by analysis using the Metal3 database^43^, and the pore helix (green), selectivity filter (yellow) and the calcium ion at site S_Ca_ of the NaKTs structure. (**b**) Superimposition of the NaKTs structure (pore helix—green; main chain atoms of selectivity filter—yellow sticks; Ca^2+^ at site S_Ca_—purple sphere) with representative motifs of PDB id 3K28 and 2IZ7 (grey cartoon) with <1.0 Å RMSD and their respective Ca^2+^ ions are shown in green spheres.

The unusual helix-turn Ca^2+^-binding motif forming the S_Ca_ site is a structural feature that has not been previously observed in other crystallographic structures of tetrameric ion channels. Interestingly, a large number of similar helix-turn Ca^2+^-binding motifs could be identified from a search via the Metal3 webserver^28^ (Fig. 6). Interestingly, Ca^2+^ appears to be functionally important for several systems where the helix-turn motif is found, for example, it triggers the assembly process of SbsB, a layer-forming component of the cell-wall in archaea and many bacteria^29^, it is essential for the enzyme activation of human transglutaminase 3, and it is required for the toxin activity of *Clostridium botulinum* neurotoxins^30^. It should be noted, however, that a large fraction of the structures with a helix-turn binding motif include coordination by negatively charged side chains, which are absent from the S_Ca_ site of the NaKTs channel. As a result, the ion binding affinity to S_Ca_ is likely to be weak. Weak ion binding affinity is generally thought to be consistent with the function of a channel, as strong binding would oppose rapid conduction. While the role of Ca^2+^ in NaKTs is not as a permeant ion but rather to activate the channel, low binding affinity for the site S_Ca_ is nonetheless reasonable, as it is accessible via the selectivity filter. In the case of the layer-forming protein SbsB (PDB id 4AQ1), the measured *K*_1/2_ falls within a range of 0.9–110 μM, a value that is considerably smaller than the *K*_1/2_ of ∼4 mM determined for the NaKTs channel on the basis of the open-channel probability (Fig. 3f).

### MD simulations

**Figure 7:**
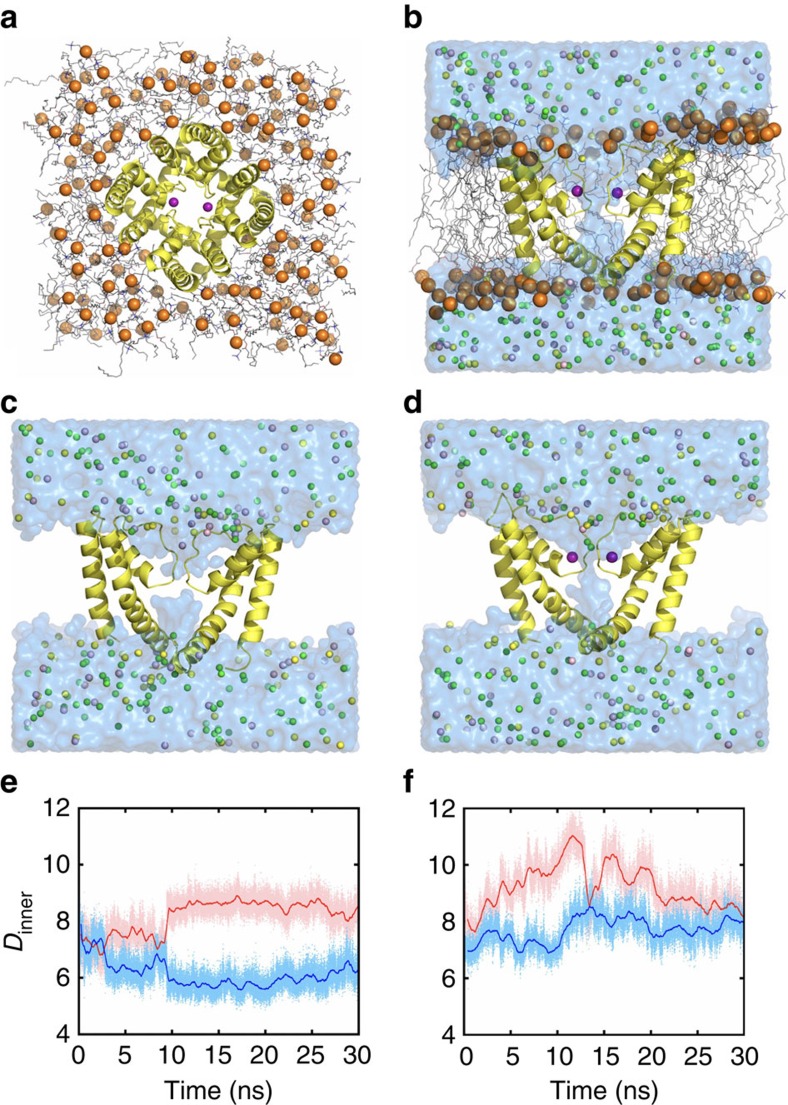
MD simulations. (**a**) The top view and (**b**) the side view of the two Ca^2+^ bound membrane-channel system before simulations using the Drude polarizable force field. After 30 ns simulation, the pore closes in the system (**c**) without any Ca^2+^ at the S_Ca_ site, whereas the selectivity filter pore remains open and permeable to water in the system (**d**) with two Ca^2+^ bound at the S_Ca_ site. The NaKTs channel is shown in yellow cartoon presentation with the front and back subunits hidden in (**b**–**d**) for the sake of clarity. POPC lipids are shown as grey lines with their phosphates in orange spheres. Ca^2+^ ions are indicated by purple spheres. The other ions including K^+^ (light pink), Na^+^ (yellow) and Cl^−^ (green) are shown as well. Surface rendering (marine) is used to show the water accessible regions in the system. The molecular presentation is created using PyMOL. (**e**,**f**) show the time series of *D*_inner_ in the zero and two S_Ca_ Ca^2+^ systems with −500 mV TM potential, respectively. The lighter-coloured points are instantaneous values of *D*_inner_ and the lines show the running averages. The two *D*_inner_ in one tetramer channel are distinguished by different colours.

To gain further insight about the structural sensitivity of the selectivity filter to the Ca^2+^ ions in the S_Ca_ binding sites, MD simulations of the NaKTs channel tetramer (ABDE) embedded in fully hydrated POPC bilayer membranes were carried out. Four different systems were simulated (0, 1, 2 and 4), comprising respectively zero, one, two and four Ca^2+^ ions bound at site S_Ca_ in the selectivity filter using the polarizable Drude force field^31^,^32^,^33^,^34^,^35^ with recently refined parameters for Ca^2+^ ions^36^. Equivalent simulations carried out using the nonpolarizable additive CHARMM36 (C36) force field^37^,^38^ were unstable (See METHODS for details). The system with two Ca^2+^ at the S_Ca_ site is shown in Fig. 7a,b. The simulations with double Ca^2+^ occupancy display relatively small deviation from the crystal structure when compared with the other systems (Supplementary Fig. 6b) and also retain a water-filled selectivity filter (Fig. 7a,d). In the simulations, the number of Ca^2+^ ions bound at the S_Ca_ sites in the selectivity filter appears to control the opening of the pore. This is observed from the distance (*D*_inner_ and *D*_outer_) between the Cα atoms (Fig. 5) and the radius profile of the pore restriction zone (R_min_), which is near residue Pro63 at the intracellular end of the filter (Supplementary Fig. 6c). In the simulations, the diameter measured across the pore between the diagonally placed Pro63 (*D*_inner_ in Fig. 5) increases as the S_Ca_ sites become occupied by Ca^2+^. This trend continues until two of the S_Ca_ sites are filled with Ca^2+^. Further increasing the number of Ca^2+^ at the S_Ca_ sites does not lead to a more open pore. Instead, it causes structural distortions of the filter and closure of the intracellular gate formed by M2 (Supplementary Fig. 6a). The R_min_ profiles in Supplementary Fig. 6c corroborate the notion that Ca^2+^ binding at the S_Ca_ sites promotes the opening of the selectivity filter. Increasing the number of Ca^2+^ ions in the selectivity filter from zero to two leads to more and longer incidents of transient pore openings (Supplementary Fig. 6c). The correlation offers one explanation from a structural point of view for the relationship between [Ca^2+^] and the channel open probability *P*_o_ determined from single-channel recordings (Fig. 3f). However, no ion-conduction events were observed during the MD simulations with an applied voltage. Further analysis indicates that the unfavourable electrostatic repulsion resulting from Ca^2+^ ion bound in the sites S_ca_ likely blocks the passage of other cations through the selectivity filter.

## Discussion

Knowledge of the landscape of conformations accessible to the selectivity filter of tetrameric K^+^ channels remains limited. To make further progress, we present here a first characterization of a novel tetrameric ion channel, referred to as NaKTs, with an unusual proline-rich selectivity filter sequence using X-ray crystallography, electrophysiology and MD simulations. Sequence comparison suggests that, despite the differences, this channel is closely related to the members of the broad family of tetrameric 2-TM channels. The NaKTs channel was crystallized with its intracellular gate in an open state. The X-ray structures revealed the presence of Ca^2+^ binding sites, S_Ca,_ formed by main chain atoms of residues Pro63, Gly65, Ser59 and Leu62, near the intracellular end of the selectivity filter. A database search reveals that similar helix-turn Ca^2+^-binding motifs are found in multiple proteins, with a broad variation in the type of coordinating ligands involved. Single-channel recordings demonstrate that the NaKTs channel is a Ca^2+^-activated non-selective cation channel. Analysis of crystallographic data indicates that the presence of bound Ca^2+^ at diagonally-opposing sites inside the selectivity filter controls the diameter of the conducting pore. Pro63, which contributes to the S_Ca_ binding site, appears to be a key residue responsible for the channel activation triggered by the binding of Ca^2+^. The direct functional implication of the S_Ca_ binding site on channel activation is confirmed by engineering a chimera protein in which the classic selectivity filter of the KcsA channel has been inserted in the NaKTs channel. While no channel activity is observed with the wild-type NaKTs unless Ca^2+^ is present (Fig. 3e), the chimeric NaKTs-SF_KcsA_ channel conducts K^+^ in the absence of any Ca^2+^ (Fig. 3g). The importance of Pro63 is supported by the results from MD simulations with a polarizable force field. The results from MD strengthen the view that the Ca^2+^ binding motif located at the base of the selectivity filter may act as an activating switch.

Paradoxically, even though the Ca^2+^ bound in the site S_Ca_ kept the selectivity filter in the crystallographic open state, no ion-conduction events were observed during the MD simulations with an applied voltage due to the unfavourable electrostatic repulsion from the bound Ca^2+^. It is also possible that the binding of Ca^2+^ could induce additional conformational changes not yet observed, or that the X-ray structure obtained from a chymotrypsin-treated truncated form of the protein is not fully representative of the channel probed by the electrophysiological experiments. For example, the intracellular gate of KcsA truncated is more wide-open than KcsA full-length^17^,^39^. The situation may be different in the case of the NaKTs channel, as the conductance of the truncated channel (Fig. 3a) is smaller than that of the full-length channel (Supplementary Fig. 7a). To be as consistent as possible with the crystal structure, the functional experiments were carried out with the truncated chymotrypsin-digested form of the NaKTs channel. However, additional experiments with the full-length channel NaKTs channel indicate that it exhibits a complex functional behaviour, with two major conductance levels. Furthermore, the full-length channel undergoes some degradation over time at room temperature, which may partly explain its complex functional behaviour. The smaller conductance level is observed more frequently with the truncated form of the channel while the larger level occurred more frequently with the full-length form of the channel (Supplementary Fig. 7a). Nonetheless, while the truncated form of the channel displays a more reliable functional behaviour than the full-length form, it remains somewhat complex (Supplementary Fig. 7b). It is also noticeable that the kinetic behaviour of the full-length channel is slower than that of the truncated channel. These observations suggest that there might be two open-channel conformations, and their relative probability is different in the chymotrypsin-digested and full-length form of the channel.

While Ca^2+^ binding at the site S_Ca_ is key for channel activation, a complex interplay between gating and conduction might take place because the permeating cations and Ca^2+^ must translocate through the same passageway. This suggests various mechanistic scenarios whereby the occurrence of ‘open' conformations of the pore could be simultaneously promoted by the reversible binding of Ca^2+^ as well as blocked for ion conduction. In other words, the channel could display sustained periods of conduction if the lifetime of the open state persists for sufficiently long time after the dissociation of the activating Ca^2+^ ion(s). The intriguing mechanism by which Ca^2+^-activation and ion conduction are interrelated in the NaKTs channel will require further investigation.

In closing, while the primary objective of the present study was to characterize the structure and function of a novel tetrameric 2-TM cationic channel in relation to other K^+^ channels, the striking structural differences presented by the pore of the NaKTs channel (its uncommon proline-rich sequence LPMGNGPLSP is not found in human channels) could potentially offer a useful target for the discovery of specific inhibitors that may be of therapeutic interest. *Tsukamurella* species are Gram-positive bacilli that have been isolated from environmental samples and sludge, that can lead to human diseases^40^. Catheter related bloodstream infections (CR-BSI), the most common presentation of *Tsukamurella* species infections, primarily occur in patients with indwelling central lines and/or immune suppression^41^. Additional clinical malfunctions include bacteremia^42^,^43^, meningitis^44^, peritonitis^45^, and cutaneous, lung and knee prosthesis infection^43^,^46^. *Tsukamurella* species is also recognized as a potential pathogen in immunocompromised patients^47^.

## Methods

### Expression and purification

Genomic DNA from *T. paurometabola* (DSM 20,162) was obtained from the DSMZ (German Collection of Microorganisms and Cell Cultures, Braunschweig, Germany). The genomic databases search leading to the identification of a 2-TM tetrameric channel from *T. paurometabola* used PsiBlast^48^, followed by manual analysis to focus on the sequence in the selectivity filter region. The *Tpau_1687* gene encoding residues 1–123 of an ion transport two domain protein was amplified by PCR and cloned into the pQE60 expression vector (Qiagen). The primers used to clone the full-length NaKTs channel into pQE60 used the NcoI and BamHI sites of pQE60. The forward primer is 5′-CGTATA**ACATGT**TGGGTCTCACATTGATGTTCAAGAGATTC-3′ and the reverse primer is: 5′-CTTGTA**GGATCC**GTCCTCCGCCTCGGCCGAAC-3′ (restriction sites highlighted in bold and the NaKTs coding sequence is underlined). The NaKTs ion channel protein was expressed in *E. coli* strain XL-1 Blue cells on induction with 0.5 mM isopropyl-β-D-thiogalactopyranoside (IPTG) at 37 °C for 3 h at an OD_600_ of 0.6–0.8. Cell pellets were resuspended in buffer A (20 mM Tris, pH 8; 0.2 M NaCl) plus 1 mM PMSF and DNase I. Cells were lysed by homogenization. The cell lysate was clarified by centrifugation at 107,000*g* for 35 min at 4 °C. and the membrane pellet was solubilized by incubation at 4 °C in Buffer B (Buffer A plus 10 mM DDM (*n*-Dodecyl-β-D-maltoside) or 40 mM DM (*n*-Decyl-β-D-maltoside). The supernatant was then purified using a cobalt metal affinity column pre-washed with buffer A and 0.5 mM β-DDM. The affinity-bound protein was eluted with Buffer A containing 500 mM imidazole and 0.5 mM β-DDM. The 6 × His C-terminal tag along with the unstable cytoplasmic domain was removed from the eluted protein by incubation with chymotrypsin for 2 h at room temperature. The cleaved protein was further purified by gel filtration (buffer: 20 mM HEPES pH 8, 200 mM NaCl and 0.3 mM β-DDM). The peak fraction was concentrated to 8–25 mg ml^−1^ and used for crystallization. To confirm the role of the S_Ca_ binding site, a chimera NaKTs-SF_KcsA_ channel was engineered and purified, where the selectivity filter of NaKTs (PMGNGP) was replaced by the corresponding residues from the selectivity filter of KcsA (TVGYGD) using QuikChange Site-Directed Mutagenesis (Agilent Technologies). The primers used to make the NaKTs-SF_KcsA_ chimera are as follow. To first make a NaKTs without a selectivity filter (NaKTS-noSF), the forward primer is 5′-CTCGGTGGGATTGCTCAGCCCCACGC-3′ and the reverse primer is 5′-GCGTGGGGCTGAGCAATCCCACCGAG-3′. To insert the KcsA selectivity filter into NaKTs-noSF, the forward primer is 5′-CTCGGTGGGATTG**ACCGTCGGCTACGGCGAC**CTCAGCCCCACGC-3′ and the reverse primer is 5′-GCGTGGGGCTGAG**GTCGCCGTAGCCGACGGT**CAATCCCACCGAG-′3 (insertion in bold). The NaKTs-SF_KcsA_ chimera was expressed and purified according to the same protocol as the wild-type protein.

### Crystallization and data collection

Trials were carried out in the presence and absence of 5 mM CaCl_2_ in sitting drop 96 well, 3 drop crystallization plates (Hampton Research), using various commercially available crystallization kits (JCSG+ suite (Qiagen), Wizard I&II (Emerald Bio), HR2 110, HR2 112, HR2 136, PEG I & II (Hampton Research), HTS Screen II (Jena Biosciences)). Crystals were obtained in JCSG+ suite conditions 18 (50% PEG 200/5% PEG 1,000; 0.1 M phosphate/citrate, pH 4.2) and 22 (50% PEG 200, 0.2 M MgCl_2_, 0.1 M sodium cacodylate, pH 6.5). More than 150 crystals obtained at varying conditions were screened at −180 °C under a nitrogen stream. All datasets were collected on the Northeastern Collaborative Access Team beamline NECAT 24-ID-C at Advanced Photon Source (APS) of the Argonne National Laboratory (ANL) on a PILATUS 6MF detector at a wavelength of 0.97920 Å. Chymotrypsin-digested NaKTs ion channel crystals obtained in JCSG+ suite condition 18 diffracted only to 7 Å resolution. The best achievable diffraction data (3.15 Å resolution) were obtained in JCSG+ condition 22. Three different crystallographic datasets were obtained at 3.15 Å (dataset 2), 3.36 Å (dataset 3) and 3.6 Å (dataset 1) resolution and the data were processed using the HKL2000 suite^49^; see Table 1. NaKTs ion channels crystallized in the I4 space group and contained 6 molecules per asymmetric unit.

### Structural refinement

Three datasets were collected, labelled chronologically 1, 2 and 3, at 3.6, 3.15 and 3.4 Å resolution, respectively. For each dataset, the asymmetric unit contains six distinct subunits (monomers labelled A, B, C, D, E, F), which can be used to generate three complete tetramers: Tetramer 1 is formed by subunits A, B, D and E; tetramer 2 is formed by chain C and chains G, H and I generated by crystallographic fourfold symmetry from chain C; tetramer 3 is formed by chain F and chains J, K and L generated by crystallographic fourfold symmetry from chain F. For dataset 2 (PDB ID: 5CBG), the structure solution was determined by molecular replacement with PHASER in the PHENIX program^50^ using the pore helix (residues 49–61) of NaK (PDB ID: 2AHY) structure as an initial model. Automated model building was performed using the PHENIX-Autobuild program and repeated cycles of model building were done using COOT^51^. Structure refinement was done using REFMAC 5.5 in the CCP4 suite of programs^52^. Calcium ions were located based on *F*_o_−*F*_c_ difference map peak at 4.5σ cutoff and water molecules at 3σ cutoff. The water molecules were also located based on the Coot:find water facility in REFMAC (CCP4 6.5.001) and finally checked manually using the electron density map in COOT^51^. The N-terminal residues 1–4 and C-terminal residues 107–123 were disordered. The final model contains six monomers (A, B, C, D, E and F) of residues 5–106 and 8 Ca^2+^ ions refined to *R*_cryst_ and *R*_free_ of 23.6 and 29.3%, respectively, with no outliers in disallowed regions of the Ramachandran plot. Two additional crystal structures, at 3.4 Å (dataset 3) and 3.6 Å (dataset 1) resolution, were determined by molecular replacement, using the NaKTs 3.15 Å crystal structure as an initial search model followed by repeated cycles of model building in COOT and refinement with REFMAC 5.5 in the CCP4 suite of programs. For dataset 3 (PDB ID: 5CBH), the N-terminal residues 1–4 and C-terminal residues 107–123 were disordered in the crystal structure. The final model contains six monomers (A, B, C, D, E and F) of residues 5–106 and 8 Ca^2+^ ions refined to *R*_cryst_ and *R*_free_ of 23.2 and 26.2%, respectively, with no outliers in disallowed regions of the Ramachandran plot. Tetramer 1 (Chain ABDE) contains four Ca^2+^ ions located at site S_Ca_ and two Ca^2+^ ions at sites 2 and 3. Tetramer 2 (Chain CGHI) has no ions in the pore. Tetramer 3 (Chain FJKL) contains two Ca^2+^ ions located at sites S0 and 1. For dataset 1 (PDB ID: 5CBH), the N-terminal residues 1–4 and C-terminal residues 107–123 were disordered in the crystal structure. The final model contains six monomers (A, B, C, D, E and F) of residues 5–106 and three Ca^2+^ ions and refined to *R*_cryst_ and *R*_free_ of 23.87 and 27.9%, respectively, with no outliers in disallowed regions of the Ramachandran plot. Tetramer 1 (Chain ABDE) contains two Ca^2+^ ions bound diagonally opposite to each other at site S_Ca_ and 1 Ca^2+^ ion bound at site 2. Tetramer 2 (Chain CGHI) and Tetramer 3 (Chain FJKL) have no ions in the pore.

### Determination of calcium ions

Calcium ions (Ca^2+^) were identified based on the *F*_o_−*F*_c_ map at 4.5σ cutoff. A cluster of Ca^2+^ ions was observed at the centre of the hexamer of tetramer assembly (overall molecular packing). Calcium positions in all of the three crystal structures were initially identified based on the *F*_o_−*F*_c_ difference map peak at 4.5σ cutoff and confirmed by calculating an anomalous density map using SAD phasing in CNS program^53^,^54^. Anomalous maps were calculated by calcium anomalous scattering values (*f*′=0.3002 and *f*″=0.5654) at *λ*=0.9792 Å using calcium positions obtained from the refined NaKTs ion channel structures at 3.15, 3.4 and 3.6 Å resolution and further confirmed by random anomalous peak search procedure. Anomalous density peaks >10.0σ cutoff were also observed for the cluster of calcium ions located at the centre of the hexamer of tetramer assembly. The calcium ion sub-structure occupancy refinement was performed using SAD data in REFMAC 5.5 in the CCP4 suite of programs. The calcium ion occupancy along the ion-conduction pathway and their corresponding B-factor values are reported in Supplementary Table 2. All structure figures were generated in PyMOL (http://www.pymol.org/).

### Functional measurements

All single-channel recording and analysis experiments were carried out using Port-a-Patch patch clamp system (Nanion Technologies GmbH, Munich, Germany). Planar lipid bilayers were obtained from GUVs prepared by using the electroformation method^55^ in an indium tin oxide (ITO) coated glass chamber connected to the Nanion Vesicle Prep Pro setup (Nanion Technologies GmbH, Munich, Germany). A mixture of 10 mM DPhPC (2-diphytanoyl-sn-glycero-3-Phosphocholine, Avanti Polar Lipids Inc.) and 1 mM cholesterol, dissolved in chloroform, was deposited on the ITO-coated glass surface and air-dried. After complete evaporation of the solvent, the lipid film was rehydrated with 210 μl of 1 M sorbitol. GUVs were formed by electroswelling using an alternating electric field of amplitude 3 V, 5 Hz for 2 h. GUVs were collected and used directly for the reconstitution of the ion channel. Purified chymotrypsin-treated NaKTs channel was reconstituted into the GUVs solution to a final concentration of 40 μg ml^−1^. Giga-ohmic seals were formed within a few seconds by pipetting 5 μl of the protein-GUVs on the micro-structured glass patch clamp NPC-1 Chips by Nanion. Channel activity was recorded under symmetric conditions (unless otherwise stated) using 20 mM HEPES, pH 7.5, 0.2 M NaCl/KCl as the internal solution and 20 mM MOPS, pH 4, 0.2 M NaCl/KCl, as the external solution. The channel is active only at pH 4 (MOPS & HEPES); no current was detected at pH 7.5 (HEPES). The data were filtered at 3 kHz (Bessel filter, HEKA amplifier) digitized at a sampling rate of 50 kHz and analyzed with ClampFit (Axon Instruments, part of Molecular Devices, USA). Ionic currents were observed only after adding Ca^2+^ in the external solution. All *I*/*V* curves were determined using a voltage sweep procedure in which the membrane potential was changed from −40 to +40 mV in steps of 20 mV lasting 20 μs. Open-channel probability was measured as a function of Ca^2+^ concentration, with internal solution containing 20 mM HEPES, pH 7.5, 0.2 M NaCl and the external solution containing 20 mM MOPS, pH 4, 0.2 M NaCl. External CaCl_2_ was applied using a multichannel perfusion system linked to the Port-a-Patch. The membrane potential was held at −40 mV and the current recordings were measured using CaCl_2_ concentrations of 1, 2, 3, 4, 5, 7.5, 10, 12.5 and 15 mM. Single-channel currents were recorded from multiple patches for 1–3 min per Ca^2+^ concentration using continuous acquisition mode. The Po were calculated as the fraction of open time over a total time interval during which there is channel activity. For each Ca^2+^ concentration, about 3–5 separate time intervals of ∼200 ms were analyzed and the error on the Po was estimated as the mean-square deviation from the average. Single-channel recordings on the chimeric NaKTs-SF_KcsA_ channel were carried out according to the same procedure.

### Building the NaKTs channel–membrane systems

The POPC bilayers systems for MD simulations were built from the crystal structure of NaKTs using the Membrane Builder module^56^ in CHARMM-GUI^57^. The N-terminal M0 helix was not included; its inclusion resulted in water penetrating the lipid bilayers according to preliminary simulations (5 ns). The protein-membrane complex was solvated by an equal molar mixture of KCl and NaCl solutions with a cation concentration of 1 M (that is, [K^+^]=[Na^+^]=0.5 M). Each of the four protein subunits was patched with an acetylated N terminus and an amidated C terminus. The resulting system comprises ∼50,000 atoms (80 × 80 × 78 Å^3^). The ABDE tetramer in dataset 2 has two Ca^2+^ placed diagonally against each other at the S_Ca_ site. It was used to generate the double S_Ca_ occupancy system while also serving as the prototype for making the zero, single and full (that is, Ca^2+^ at all four S_Ca_ sites) S_Ca_ occupancy systems.

### Equilibration of the NaKTs channel systems

Initial equilibration simulations were performed in the NPT ensemble with constant pressure (1 atm along the *Z* axis) and temperature (300 K) with the NAMD program^58^ version 2.9. The time duration for each equilibration simulation is 10 ns. The pressure of the system was controlled by the hybrid Nosé–Hoover Langevin piston method. Langevin dynamics thermostat with a 1 ps^−1^ damping coefficient was used to keep the temperature constant. The van der Waals interactions were smoothly switched off at 10–12 Å by a switching function and the electrostatic interactions were calculated using the particle-mesh Ewald method with a mesh size of ∼1 Å. Harmonic positional restraints were applied to the protein backbone atoms and the bound Ca^2+^, keeping the structural deviation from the crystal structure to a minimum. This was necessary because it prevents the channel from closing. The force constant of the harmonic potential was set to 5 kcal mol^−1^ Å^−2^ Further equilibration of 100 ns for each system was performed on Anton with the volume and temperature of the system kept constant. Harmonic positional restraints with a force constant set to 5 kcal mol^−1^ Å^−2^ were applied to the backbone and the S_Ca_ Ca^2+^ atoms to prevent the filter from collapsing. The Nosé–Hoover thermostat was used to maintain the system temperature at 300 K^59^. The lengths of all bonds involving hydrogen atoms were constrained using M-SHAKE^60^. The cutoff of the van der Waals and short-range electrostatic interactions was set to 16.21 Å as suggested by the guesser script. Long-range electrostatic interactions were evaluated using the *k*-space Gaussian split Ewald method^61^ with a 64 × 64 × 64 mesh. The *r*-RESPA integration method^62^ was employed and long-range electrostatics were evaluated every 6 fs. The additive C36 force field^37^,^63^ was used in both the NAMD and Anton equilibration simulations.

### Simulation of the Drude model systems

After equilibration, each system was then converted to its Drude model representation using the Drude Prepper module in CHARMM-GUI^57^ with the TIP3P water molecules replaced by the polarizable water SWM4-DNP (ref. 32). The generated Drude model systems of the NaKTs channel were equilibrated using the input scripts provided in the CHARMM-GUI Drude Prepper module. The production afterwards was carried out using NAMD2.9 with the Drude polarizable model implemented^64^. The simulation protocols were analogous to those during the initial equilibration with constant pressure (1 atm along the *Z* axis) and temperature (300 K), except that a dual Langevin thermostat was used to keep the Drude particle at 1 K (ref. 64). The first 2.5 ns of each production was TM potential (*V*_mp_) free. Following it was a 30 ns production run with a *V*_mp_ of either 500 or −500 mV to investigate ion permeation. The MD simulations based on the polarizable force field were stable within a 30-ns duration. This is in stark contrast with the restraint-free NaKTs channel simulations based on the nonpolarizable force field, in which the Ca^2+^ ions at the S_Ca_ site started to dissociate in a much shorter time scale, leading to a rapid collapse of the selectivity filter.

### Simulating the hydration of pore Ca^2+^ ions

To examine the hydration structure of Ca^2+^ ions within the selectivity filter pore, four additional systems were constructed starting from the crystal structures showing four occupancy states (I to IV in Fig. 4). The setup procedure was the same as described above. Each system was equilibrated for 375 ps with reducing harmonic restraints asserted on protein heavy atoms and the Ca^2+^. At the end of the equilibration, the force constant of the harmonic restraints was 2 kcal mol^−1^ Å^−2^. It was kept the same in the 30-ns production simulations. A Langevin thermostat and a Monte Carlo barostat were used to maintain the system temperature and pressure at 300 K and 1 atm. Both the equilibration and the production were performed with the OpenMM6.2 program^65^ on GeForce GTX 780 Ti GPU processors.

### Data availability

Coordinates and structure factors have been deposited in the Protein Data Bank under accession codes 5CBF (dataset1), 5CBG (dataset2) and 5CBH (dataset3). Remaining data are available from the corresponding author upon reasonable request.

## Additional information

**How to cite this article:** Dhakshnamoorthy, B. *et al.* Structural and functional characterization of a calcium-activated cation channel from *Tsukamurella paurometabola*. *Nat. Commun.* 7:12753 doi: 10.1038/ncomms12753 (2016).

## Supplementary Material

Supplementary Information Supplementary Figures 1-7 and Supplementary Tables 1-2.

## Figures and Tables

**Table 1:** Data collection and refinement statistics.

	Dataset 1 (5CBF)	Dataset 2 (5CBG)	Dataset 3 (5CBH)
*Data collection*			
Space group	I4	I4	I4
Cell dimensions			
*a*, *b*, *c* (Å)	116.05, 116.05, 132.58	115.57, 115.57, 127.49	116.47, 116.47, 128.13
*α*, *β*, *γ* (°)	90, 90, 90	90, 90, 90	90, 90, 90
Resolution (Å)	50–3.60 (3.66–3.60)*	50–3.15 (3.20–3.15)*	50–3.37 (3.42–3.37)*
*R*_sym_ or *R*_merge_ 14.4 (75)	16 (66.5)	20.9 (86.7)	
*I*/σ*I*	3 (2.85)	3.11 (3)	3 (2.42)
Completeness (%)	99.3 (99.6)	98.7 (92.2)	96.2 (98.3)
Redundancy	4.8 (4.5)	4.9 (4.4)	3 (3.1)
			
*Refinement*			
Resolution (Å)	50–3.6	50–3.15	50–3.37
No. reflections	9,567	11,407	13,791
*R*_work_/*R*_free_	23.87 (27.9)	23.68 (29.8)	23.22 (26.2)
No. atoms	4,627	4,771	4,633
Protein	4,620	4,620	4,620
Detergent/Ca^2+^	0/3	99/8	0/8
Water	—	36	1
*B*-factors			
Protein	114.5	65.5	88
Detergent/ Ca^2+^	0/125	46.7/83.8	0/75.9
Water	—	38.4	8.83
R.m.s. deviations			
Bond lengths (Å)	0.015	0.013	0.014
Bond angles (°)	2.23	1.88	1.95

R.m.s., root-mean-square.

^*^Values in parentheses are for highest-resolution shell.
