# Supplementary material for: Structural and functional characterization of a calcium-activated cation channel from Tsukamurella paurometabola
Source: Nat Commun. 2016 Sep 28;7:12753. doi: 10.1038/ncomms12753 (PMC5052707; doi:10.1038/ncomms12753)
Supplement: Supplementary Information — Supplementary Figures 1-7 and Supplementary Tables 1-2. [file ncomms12753-s1.pdf]

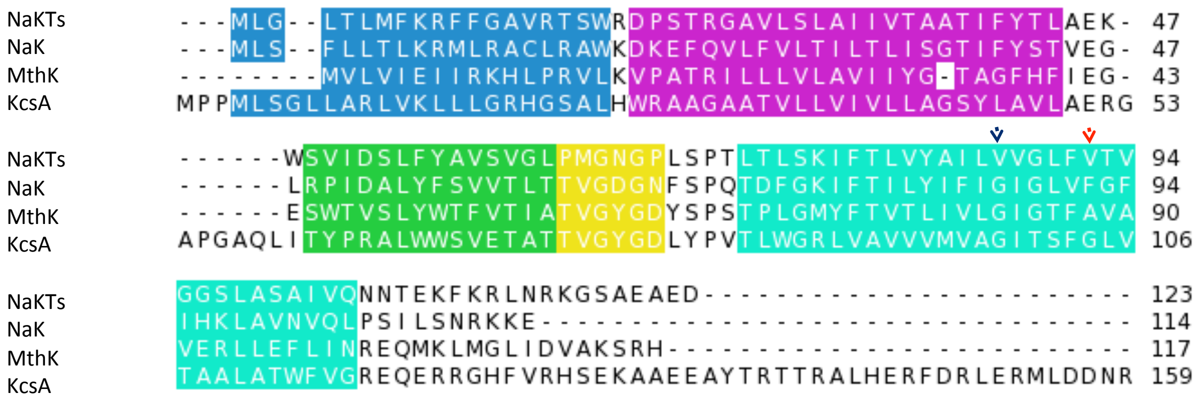

Supplementary Figure 1. Multiple sequence alignment of the NaKTs ion channel along with, NaK, MthK and KcsA sequences. The inner helix bending occurring at Val87 in the NaKTs ion channel aligned with rest of the channels is indicated by a blue arrow. The constriction zone residue Val92 in the NaKTs sequence aligned with the rest of the channels is indicated by a red arrow.

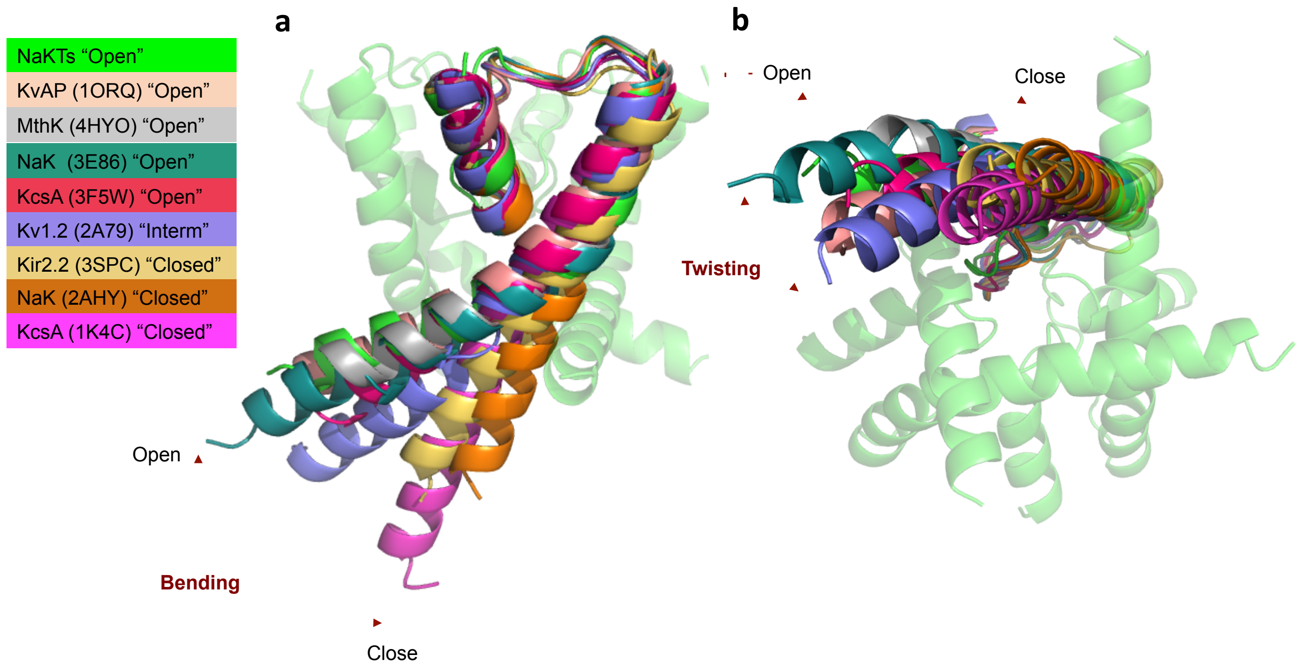

Supplementary Figure 2. Comparison of the NaKTs M2 inner helix (residues 73-102) with those from the KvAP, MthK, NaK, Kv1.2, Kir2.2, and KcsA structures. (a) shows a side view and (b) shows a bottom view. The alignment is based on the backbone atoms of the M1 helix and the pore domain.

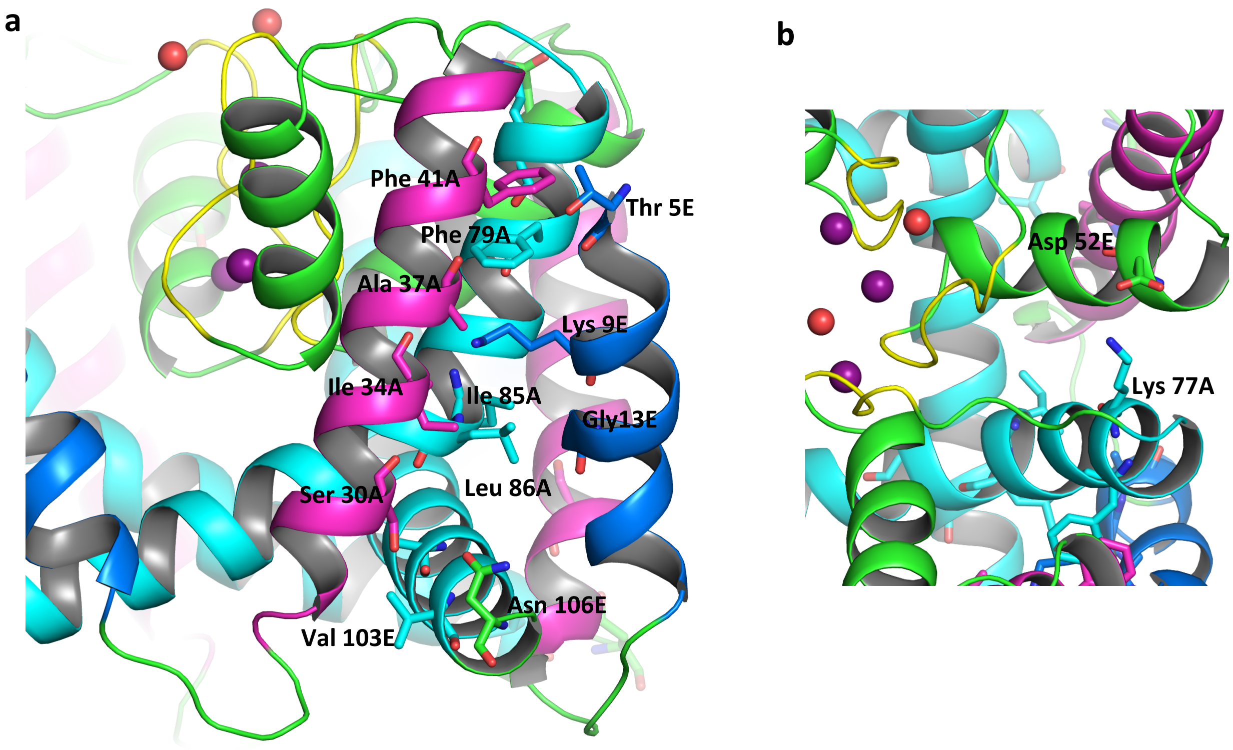

Supplementary Figure 3. (a) Intermolecular interactions near the N and C termini of the NaKTs ion channel viewed from the side. **(b)** Intermolecular interaction between residue Asp52 in pore helix of subunit E and Lys 77 of the neighboring subunit A, viewed from the top. M0 helix is shown in blue, M1 helix is shown in magenta, pore helix in green, selectivity filter is shown in yellow, M2 helix is shown in cyan and the loop regions are shown in green. Calcium ions and water molecules are shown as purple and red spheres respectively.

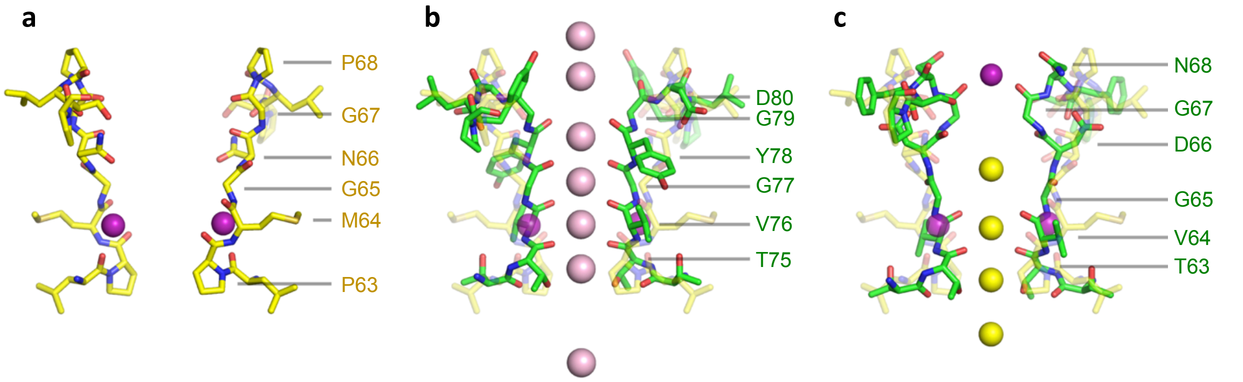

Supplementary Figure 4. Selectivity filters of **(a)** the NaKTs channel in yellow, **(b)** KcsA in green (PDBID 1K4C), and **(c)** the NaK channel in green (PDBID 2AHY). Ca^{2+} (purple), K^+ (light pink), and Na^+ (yellow) ions are as spheres. The filters are shown in cartoon representation. The KcsA and NaK channel filters are aligned to that of the NaKTs channel using backbone atoms.

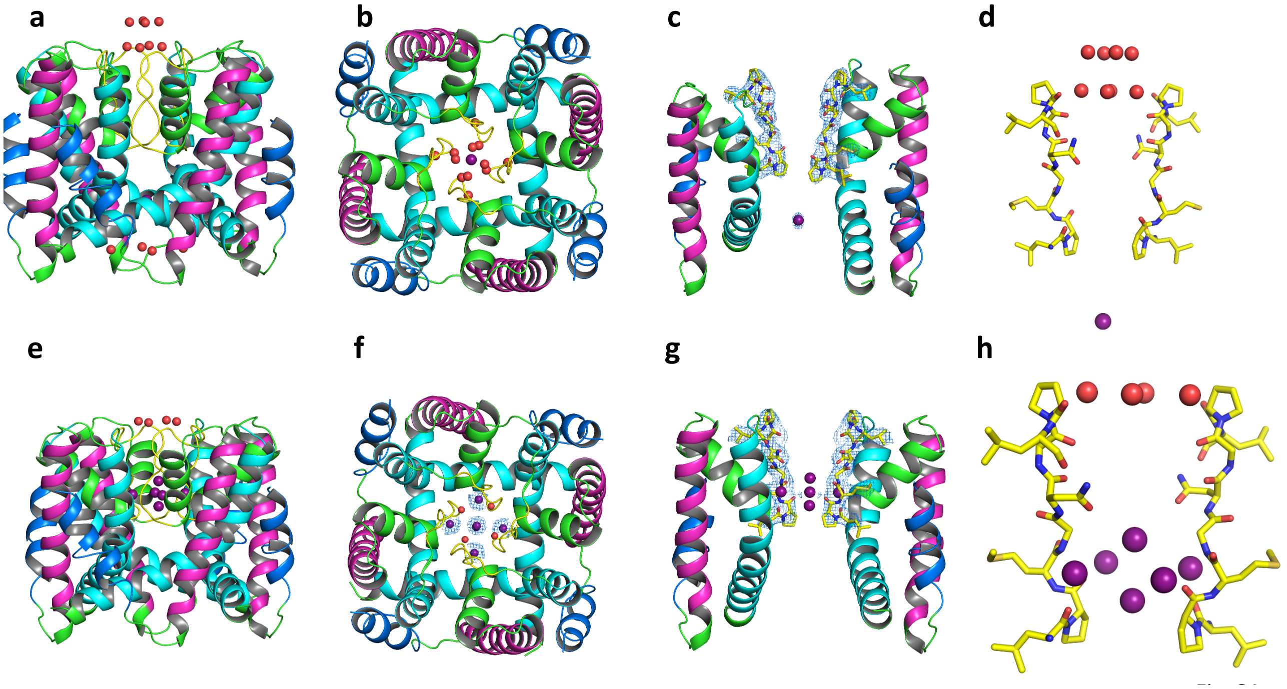

Supplementary Figure 5. X-ray crystal structure of the NaKTs ion channel determined at 3.15Å resolution (dataset 2). M0 helix (residues 5-19) is shown in blue, M1 inner helix (residues 25-45) is shown in magenta, pore helix (residues 46-61) is shown in green, selectivity filter (residues 62-69) is shown in yellow, M2 outer helix is shown in cyan and the loop regions are shown in green. Calcium ions and water molecules are shown as purple and red spheres respectively. **(a & b)** Cartoon representation of side & top view of the NaKTs tetramer (chain CGHI) with bound Ca^{2+} ions and water molecules. **(c)** The transmembrane sections of the two opposing monomers of the NaKTs ion channel protein (chain C & chain H) are shown with the $2F_o$-F_c density map at 1.0σ contour for the selectivity filter region (yellow sticks). **(d)** A close-up view of the NaKTs selectivity filter with bound Ca^{2+} ions and water molecules. **(e)** Cartoon representation of side view of the NaKTs tetramer (chain FJKL) with bound Ca^{2+} ion at the internal vestibule and water molecules at the extracellular surface. **(f)** Top view of the NaKTs tetramer (chain FJKL). Bound Ca^{2+} ions and water molecules are shown with the $2F_o$-F_c density map at 2.0σ contour for the Ca^{2+} ions. **(g)** The transmembrane sections of the two opposing monomers of the NaKTs ion channel protein (chain F & chain K) are shown with the $2F_o$-F_c density map at 1.0σ contour for the selectivity filter region (yellow sticks). **(h)** A close-up view of the NaKTs selectivity filter (chain FJKL) with bound Ca^{2+} ions and water molecules.

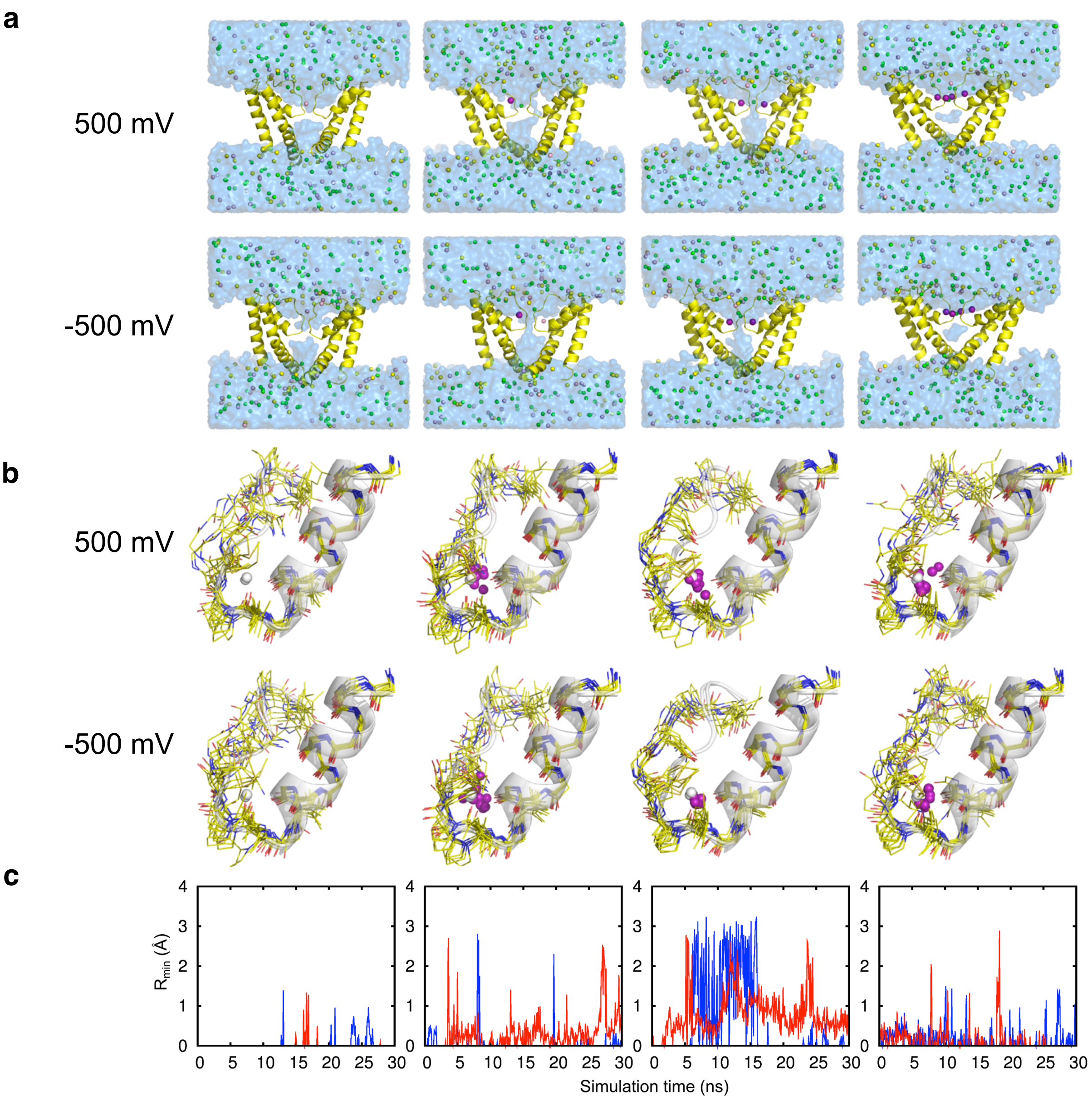

Supplementary Figure 6. (A) Snapshots from the Drude model system simulations show the water filled NaKTs channel lumen. The snapshots are taken at the 30-ns time mark. The protein is shown in cartoon representation and the ions are shown as spheres with Ca^{2+} in purple, K^{+} in light pink, Na^{+} in yellow, and Cl^{-} in green. Water is shown in surface presentation. (B) Overlap of the pore helix and selectivity filters from the simulation snapshots and the X-ray structure. 10 snapshots from the Drude system simulations are randomly chosen. The pore helix and the selectivity filter (yellow lines) are aligned to the X-ray structure (transparent cartoon) using the backbone atoms. Ca^{2+} ions are in purple. The crystal structure Ca^{2+} are shown as white spheres. (C) The pore constriction zone radius (R_{min}) time series from the Drude model simulations. The positive (500 mV, blue) and negative (-500 mV, red) V_{mp} systems are indicated by different colors.

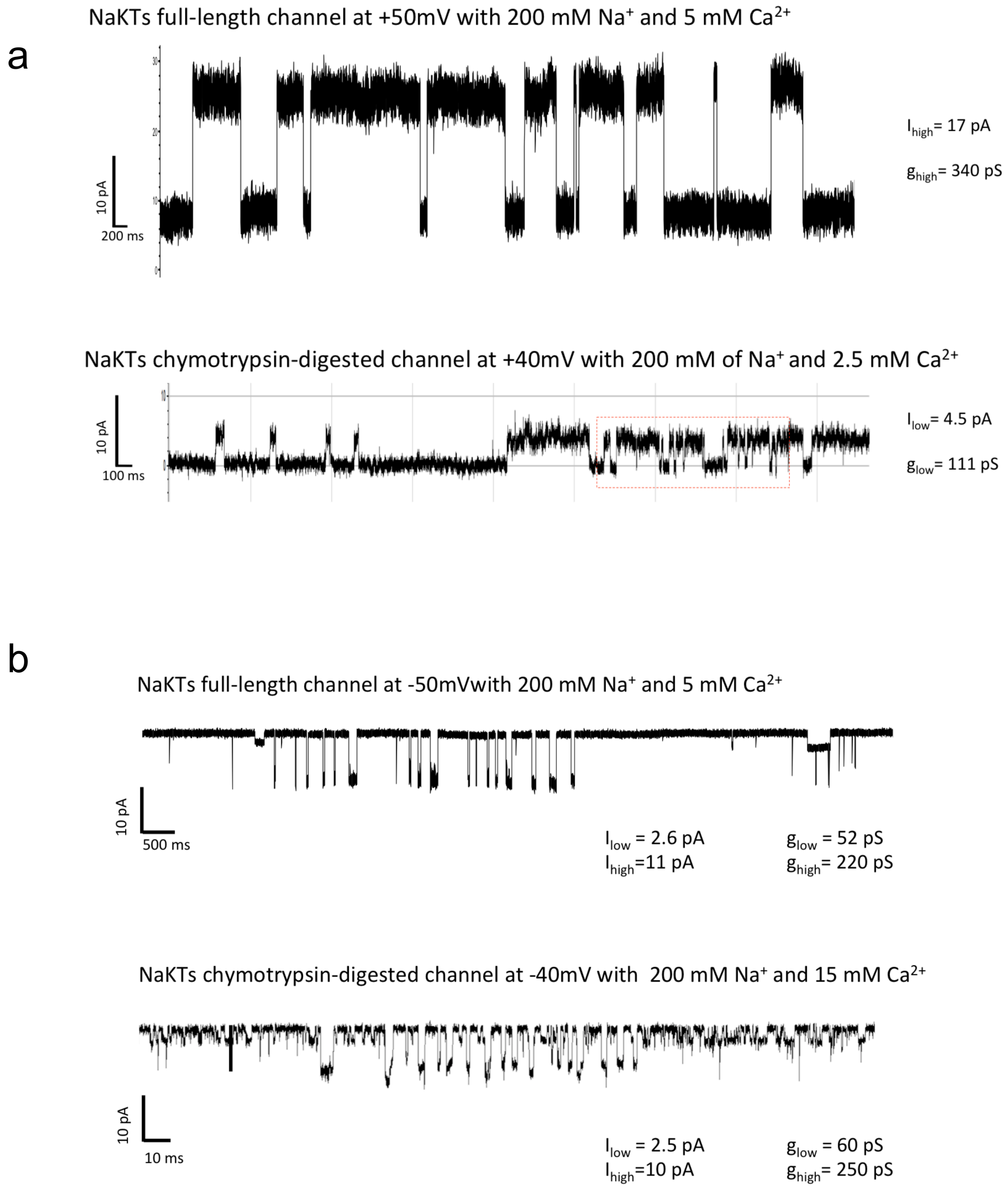

Supplementary Figure 7. Functional comparison of full-length and truncated NaKTs channel. (a) Single channel recordings for the full-length and truncated NaKTs channel. The Y-axis from ClampFit is kept to allow a direct comparison of the currents; the red box in dotted lines in shows the section of single channel recordings that is displayed in Figure 5a.While the conditions are not completely identical in the two experiments, the results are easily compared. The larger conductance level is on the order of about 250-300 pS, whereas the smaller conductance is on the order of 50-100 pS. (b) Occurrence of the two levels of conductance observed in single channel recordings of the full-length channel and truncated channel.

Supplementary Table 1. Backbone atom root mean square deviation in Å between the selectivity filters from channels within the K^+ channel family. Pairwise alignments are based on the structure of the pore helix and the selectivity filter (the PDB ID of all the structures used are given in parentheses).

	NaK (2AHY)	NaK (3E86)	MthK (4HYO)	KcsA (3F5W)	KcsA (1BL8)	KcsA (1K4C)	KcsA (1K4D)	KirBac (1P7B)	Kir2.2 (3SPC)	Kir3.2 (3SYA)	KvAP (1ORQ)	Kv1.2 (2A79)	Kv1.2/Kv2.1 (2R9R)
NaKTs	2.57	2.42	2.47	2.32	2.53	2.43	2.26	3.05	2.45	2.46	2.45	2.45	2.53
NaK (2AHY)		0.28	1.46	2.07	1.77	1.67	2.04	1.63	1.50	1.60	1.83	1.69	1.57
NaK (3E86)			1.33	1.93	1.66	1.52	1.90	1.64	1.42	1.47	1.55	1.55	1.46
MthK (4HYO)				1.17	0.52	0.40	1.02	1.45	0.37	0.72	0.53	0.50	0.31
KcsA (3F5W)					0.96	0.96	0.41	1.94	1.30	1.38	1.08	0.87	1.03
KcsA (1BL8)						0.38	0.81	1.51	0.60	0.79	0.57	0.38	0.45
KcsA (1K4C)							0.77	1.55	0.51	0.80	0.47	0.22	0.28
KcsA (1K4D)								1.84	1.10	1.22	0.91	0.70	0.88
KirBac (1P7B)									1.46	1.37	1.64	1.50	1.55
Kir2.2 (3SPC)										0.63	0.68	0.63	0.49
Kir3.2 (3SYA)											0.91	0.71	0.78
KvAP (1ORQ)												0.48	0.37
Kv1.2 (2A79)													0.35

Supplementary Table 2. **Calcium ion occupancy of the NaKTs ion channel at different states refined using SAD anomalous dataset**

**PDB ID: 5CBH (3.4Å) – Chain FJKL – State II– SAD Calcium Occupancy**			
Calcium No – State II	Ca - Site	Occupancy	B-factor (Å^2)
CA 202	S0	0.13 (Special position)	79.91
CA 201	S1	0.14 (Special position)	71.29
PDB ID: 5CBG (3.15Å) - Chain ABDE – State III – SAD Calcium Occupancy			
Calcium No – State III	Ca - Site	Occupancy	B-factor (Å^2)
CA 201	S2	0.76	55.54
CA 204	S_{Ca1}	0.95	69.41
CA 201	S_{Ca2}	0.88	62.48
PDB ID: 5CBF (3.6Å) - Chain ABDE – State III – SAD Calcium Occupancy			
Calcium No – State III	Ca - Site	Occupancy	B-factor (Å^2)
CA 202	S2	0.76	106.94
CA 201	S_{Ca1}	1.00	95.84
CA 201	S_{Ca2}	1.00	123.45
PDB ID: 5CBH (3.4Å) - Chain ABDE – State IV – SAD Calcium Occupancy			
Calcium No – State IV	Ca - Site	Occupancy	B-factor (Å^2)
CA 203	S2	0.82	65.87
CA 202	S3	0.98	140.89
CA 202	S_{Ca1}	1.00	69.84
CA 201	S_{Ca2}	0.81	83.90
CA 201	S_{Ca3}	0.86	63.02
CA 201	S_{Ca4}	0.89	85.49
PDB ID: 5CBG (3.15Å) - Chain FJKL – State IV – SAD Calcium Occupancy			
Calcium No – State IV	Ca - Site	Occupancy	B-factor (Å^2)
CA 202	S2	0.10 (Special position)	93.73
CA 205	S3	0.15(Special position)	56.58
CA 203	S_{Ca}	0.90	93.30
